# Possible association between spindle frequency and reversal-learning in aged family dogs

**DOI:** 10.1038/s41598-020-63573-9

**Published:** 2020-04-16

**Authors:** Ivaylo Borislavov Iotchev, Dóra Szabó, Anna Kis, Enikő Kubinyi

**Affiliations:** 1 Department of Ethology, Eötvös Loránd University, Budapest, Hungary; 2 Institute of Cognitive Neuroscience and Psychology, Research Centre for Natural Sciences, Budapest, Hungary

**Keywords:** Short-term memory, Neurology

## Abstract

In both humans and dogs sleep spindle occurrence between acquisition and recall of a specific memory correlate with learning performance. However, it is not known whether sleep spindle characteristics are also linked to performance beyond the span of a day, except in regard to general mental ability in humans. Such a relationship is likely, as both memory and spindle expression decline with age in both species (in dogs specifically the density and amplitude of slow spindles). We investigated if spindle amplitude, density (spindles/minute) and/or frequency (waves/second) correlate with performance on a short-term memory and a reversal-learning task in old dogs (> 7 years), when measurements of behavior and EEG were on average a month apart. Higher frequencies of fast (≥ 13 Hz) spindles on the frontal and central midline electrodes, and of slow spindles (≤ 13 Hz) on the central midline electrode were linked to worse performance on a reversal-learning task. The present findings suggest a role for spindle frequency as a biomarker of cognitive aging across species: Changes in spindle frequency are associated with dementia risk and onset in humans and declining learning performance in the dog.

## Introduction

Sleep spindles are brief trains of rhythmic activity, at least half a second in duration^1^ and maximally 6 seconds long^2^, which appear in the EEG signal of humans^3,4^ and other mammals^5^ during non-REM sleep, in particular stage 2 of non-REM sleep in humans. They are commonly distinguished in a slow (predominantly frontal, ≤ 13 Hz) and fast (≥ 13 Hz, predominantly central and posterior) subtype^6^.

One promising model animal in comparative sleep spindle research is the dog (*Canis familiaris*). A shared anthropogenic environment and evolutionary adaptation to its dynamics^7^ characterize dogs as an animal model for human conditions in general^8^, and they have specifically been argued a favorable model in comparative neuroscience as well^9^. Moreover, there is recent evidence^10,11^ that, in dogs, transients oscillating in the 9–16 Hz frequency range, corresponding to the broad definition of the sigma band or spindling frequency in humans^12,13^, are analogous to human sleep spindles (See Table 1 for an overview of these analogies and the associated literature).

**Table 1:** Overview of how sleep spindle features are affected in humans and dogs by age, sex and exposure to learning tasks.

Spindle characteristic	Phenotype	Correlation	Human references	Dog references
**OCCURRENCE**				
Spindle occurrence	Post-sleep improvement on novel tasks	Positive	Clemens *et al*. (2005; 2006), Cox *et al*. (2012), Gais *et al*. (2002)	Iotchev *et al*. (2017)
Spindle occurrence	Learning	Higher with exposure to new information	Gais *et al*. (2002), Schabus *et al*. (2008), Schmidt *et al*. (2006)	Iotchev *et al*. (2017)
Spindle occurrence	Aging	Negative	Martin *et al*. (2012), Crowley *et al*. (2002), Guazzelli *et al*. (1986)	Iotchev *et al*. (2019)*
Slow (≤ 13 Hz) spindle occurrence	Verbal/abstract learning	Positive	Clemens *et al*. (2005), Schmidt *et al*. (2006), Schabus *et al*. (2008)	Iotchev *et al*. (2017)
Fast (≥ 13 Hz) spindle occurrence	Sexual dimorphism	Higher in women	Gaillard & Blois (1981), Ackermann *et al*. (2015)	Iotchev *et al*. (2017; 2019)
Fast (≥ 13 Hz) spindle occurrence	Topography	Higher over central and posterior derivations	Gibbs & Gibbs (1961)	Iotchev *et al*. (2019)
Fast (≥ 13 Hz) spindle occurrence	Menstrual cycle	Fluctuates	Driver *et al*. (1996), Baker *et al*. (2007), de Zambotti *et al*. (2015)	Iotchev *et al*. (2019)**
**AMPLITUDE**				
Spindle amplitude	IQ	Positive	Ujma (2018)	none
Frontal, slow (≤ 13 Hz) spindle amplitude	Aging	Negative	Martin *et al*. (2012), Landolt & Borbély (2001)	Iotchev *et al*. (2019)
Fast (≥ 13 Hz) spindle amplitude	Sexual dimorphism	Higher in women	Ujma *et al*. (2014), Bódizs (2017)	Iotchev *et al*. (2019)
**FREQUENCY**				
Spindle frequency	Aging, dementia	Higher in elderly/patients	Crowley *et al*. (2002), Principe & Smith (1982), Ktonas *et al*. (2007)	Iotchev *et al*. (2019)
Fast (≥ 13 Hz) spindle frequency	Sexual dimorphism	Higher in women	Driver *et al*. (1996), Bódizs (2017)	Iotchev *et al*. (2019)
**DURATION**				
Spindle duration	IQ	Positive	Ujma (2018)	none
Spindle duration	Aging	Negative	Guazzelli *et al*. (1986), Crowley *et al*. (2002)	none

The two studies in the dog are based on the same detection methods. * = in dogs only for central, slow spindles; ** = menstrual fluctuation of spindle features, like fast spindle occurrence and frequency were not demonstrated directly in the dog, rather it was found that differences in fast spindle expression between the sexes are specific to sexually intact animals and that these features varied strongly between intact females which implied an effect of hormonal variation.

As a note of caution, regarding spindle-cognition associations in general, detection methods for sleep spindles tend to correlate poorly with each other^14,15^ which is challenging to the comparability between studies. In addition, sleep spindles also appear to display a link to memory consolidation and learning only under specific circumstances. The distance of the to-be-learned material to prior knowledge^16,17^, the exact stage of non-REM sleep in which spindles are measured^18^ and the timing relative to cortical up-states and ripples^17–19^ all seem to have an influence. In light of this it is not surprising that associations between sleep spindles and learning are not always replicated^20^. Considering the controversy about the comparability of different spindle detection methods^14,15^, it is important to note that both studies to date on spindle expression in dogs (Iotchev *et al*.^10,11^) used the same search criteria. The two studies are therefore directly comparable with each other and the present study. These dog spindle investigations have replicated several findings in the human literature (Table 1), although dog sleep spindles also display some unique dynamics. In particular frontal, fast (≥ 13 Hz) spindles do not decline, but increase with age in the dog.

Age seems to affect both memory^21–24^ and the expression of sleep spindles^11,25–29^ in humans and dogs, but it has seldom been investigated in either species how changes across time in one variable associates with changes in the other. Attempts thus far have either failed to show this type of association^28^ or entirely omitted difference score analyses. Even when spindle activity (e.g. density, amplitude) was recorded at different time points or across age-groups it was only compared with immediate post-sleep behavior^30–32^ (the examples include research on early development and IQ). A third common line of research has revealed associations between changes in spindle characteristics and the risk for developing dementia, however the syndrome was not broken down into specific domains (e.g. memory or problem solving decline)^33,34^ so that it is not known which functions specifically change as spindle expression changes.

Day-to-day fluctuations in spindle occurrence and frequency may explain why difference score comparisons have mostly been avoided. Sources of such variation include the menstrual cycle^35–38^, and simple exposure to novel information, which increases spindle occurrence in humans^39–41^, rats^41,42^, and dogs^10^. It seems, therefore, that without controlling for hormonal levels and pre-sleep experience, comparing spindle activity and memory performance measured further away than a day from each other could be problematic. On the other hand, relatively stable spindle occurrence has been reported within individuals, across nights^43^, and the frequency ranges of spindling are also assumed to be relatively stable within individuals^13,44^. Furthermore, age-related decline of spindle occurrence and amplitude in humans^25–29^ are a convincingly replicated effect which is not masked by the expected fluctuation.

In conclusion, differences in spindle characteristics measured between widely spaced time intervals could be useful biomarkers as they more likely reflect developmental or age-related changes, but more studies need to undertake difference score comparisons to test this assumption. Moreover, although useful for studying memory consolidation, same-day correlates of memory and learning are unlikely to reflect the general performance level of the individual. This is because of the already discussed sources of day-to-day variation in, for instance, occurrence^35–37,39–41^, but also frequency^38,45^ of spindles. Same-day correlates between sleep spindles and learning are thus of low diagnostic value. It has been established that spindle amplitudes and duration in humans correlate with IQ even when each is measured more than a day apart from the other^46^. No work has investigated the same for learning and memory, and the effect sizes for IQ are also modest, though well-replicated. From a clinical point of view, it is a very important question concerning possible applications of diagnostic EEG in the veterinary praxis if a measurement can predict performance further apart in time.

In the present study we apply the same automatic spindle detection algorithm as previously used in the dog^10,11^ to a data-set containing two measurements per dog, of each EEG and behavior (a short-term memory task and a reversal-learning paradigm) to evaluate whether canine spindles are useful markers of cognitive aging. First, we investigated if changes between two widely spaced (at least 3 months apart) samples of EEG can predict changes in performance between two similarly timed measures of the two learning tasks. Second, because the interval between corresponding probes of EEG and behavior vary from several days to about a month (on average) the data also allowed us to test if any spindle features can function as markers beyond the span of a day. Finally, we focused on a sample of exclusively older dogs (minimum age = 7 years), as variation in performance in elderly individuals is likely better suited to observe markers relevant for distinguishing healthy and pathological aging. The sample from which the present selection of dogs was taken (based on the availability of EEG data) was demonstrated before to display a variability in cognitive performance^23,47^.

We hypothesized that occurrence, measured as density (spindles/minute), and/or amplitude would show a positive association with performance on the two tasks, while we expected an inverse relationship with spindle frequency, as it rises with age in humans^29,33,48^ and dogs^11^. Importantly, as done in many human studies, we separately analyzed fast and slow spindles^10,11^ as in both humans and dogs age-related changes in spindle characteristics differ between the slow and fast variety and topographically (for instance more pronounced slow spindle amplitude decline over the frontal cortex in both humans and dogs^11,25,26^).

## Methods

### Ethics statement

The behavioural observations conducted in this study complied with national and EU legislation and institutional guidelines and according to Hungarian legislation (‘1998. évi XXVIII. Törvény’ 3. §/9. — The Animal Protection Act). The Hungarian “Animal Experiments Scientific and Ethical Committee” approved the experimental procedures under the numbers: PE/EA/2019-5/2017 and PE/EA/853-2/2016. Owners provided written consent to their dogs’ participation. The information included the owner’s right to withdraw their consent at any time. Owners could at any point decline to participate with their dog and could request their data not to be used and/or deleted after collection. The study was performed in accordance with the recommendations in the International Society for Applied Ethology guidelines (www.applied-ethology.org) for the use of animals in research. Non-invasive behaviour and EEG tests are not considered as animal experiment and are therefore allowed to be conducted without any special permission from the University Institutional Animal Care and Use Committee (UIACUC). The study was performed in strict accordance with the recommendations in the International Society for Applied Ethology guidelines for the use of animals in research.

### Subjects

**Figure 1:**
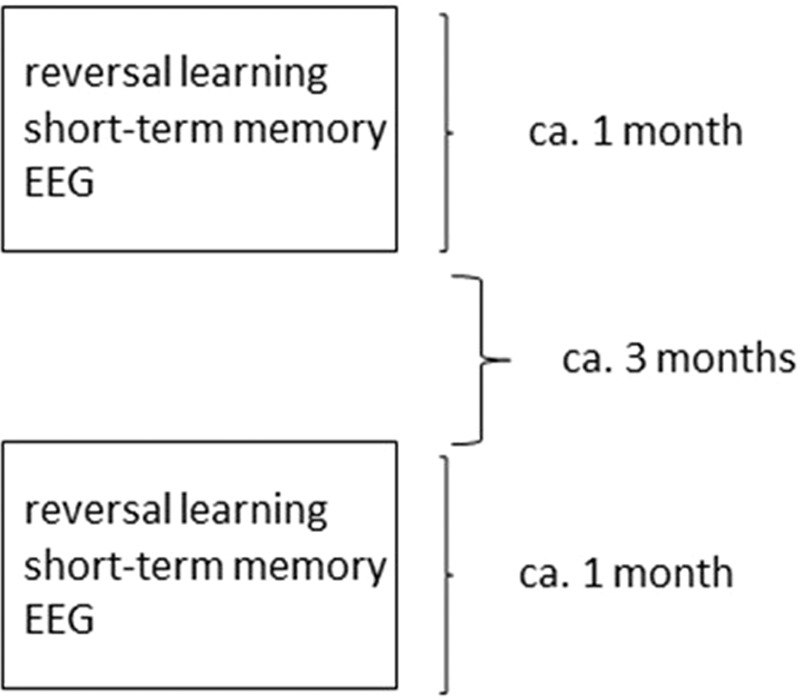
On average corresponding measures of behavior and EEG took place within one month of each other, while around at least 3 months passed from all initial to all follow-up measurements.

58 dogs, age range: 7–14 years (M ± SD = 10.3 ± 1.6), 33 females (56.9% of total sample), 49 neutered (84.5% of total sample), 28 from mixed breeds, were included in the current study, with at least one pair of corresponding measurements for each the short-term memory tests and EEG (Fig. 1). All subjects were selected to weight above 8 kg in order to minimize variation induced by lifespan differences between differently sized dog breeds.

The subjects were a sub-set of a larger data-set obtained from a longitudinal study, consisting of an array of cognitive tests (cognitive battery) used to study the effects of age^23,47^.

From all EEG recordings available for analysis (two per dog), 18 were excluded for being damaged or not containing sleep. For testing the main effects of spindle amplitude and frequency, recordings were additionally excluded if their signal contained no spindles in spite of sleep (N = 3 for slow spindles on Fz, N = 2 for slow spindles on Cz, N = 11 for fast spindles on Fz, N = 12 for fast spindles on Cz). In analyses concerning reversal-learning, subjects were also excluded if they did not attend both reversal-learning measurements (N = 7). Figure legends 3 and 4 in the result section show the final sample size in each analysis.

EEG measurements, as well as the reversal-learning and short term memory tasks, were performed on different occasions, thus the repeated design required dogs and owners to participate on six testing occasions in total. 137 ± 45.9 days (M ± SD) passed between the 1^st^ and 2^nd^ measurements of the short-term memory tests, while the distance between each short term memory and reversal-learning test was 11.6 ± 8.9 days (M ± SD). The distance between the first EEG and first test of short-term memory, as well as 2^nd^ EEG and 2^nd^ memory test, was 31.2 ± 48.2 days (M ± SD), the same distances for EEG and reversal-learning: 28.4 ± 44 (M ± SD). For a schematic overview of the relative average temporal distances between the six measurements, see Fig. 1.

### Polysomnographic method

Three electrodes were placed on the skull midline. The anterior midline electrode (Fz) and central midline electrode (Cz) were referenced against Pz, which was placed on the occipital bone at the back of the dog’s head. The remaining head electrodes consisted of a ground electrode, placed on the left musculus temporalis and two additional electrodes for measuring eye movements (placed on the left and right os zygomioticum). Furthermore, electrocardiogram (ECG), respiration and muscle tone were monitored in order to aid sleep stage identification. The impedance of the active electrodes was kept below 20 kΩ. The signal was collected, pre-filtered, amplified and digitized (sampling rate: 1024 Hz/channel, SAM 25 R style MicroMed Headbox: MicroMed Inc., Houston, TX, USA). The hardware passband was set at 0.5–256 Hz, sampling rate: 512 Hz, anti-aliasing filter cut-off frequency set at 1 kHz, and 12-bit resolution covering a voltage range of ± 2 mV. The second-order software filters (high pass > 0.016 Hz, low pass <70 Hz) were implemented using System Plus Evolution software (MicroMed Inc, Houston, TX, USA).

### Spindle detection

The method used to detect spindles is described in Iotchev *et al*.^10,11^ and uses criteria proposed by Nonclercq *et al*.^44^. Importantly, the method makes use of a two-step procedure and a spectral analysis with a moving, overlapping time-window of 0.5 seconds length (the minimum duration of spindles^1^). In the first round, the algorithm ‘searches’ for time-windows, within a filtered (between 5–16 Hz) non-REM signal, in which the maximum power is in the sigma range (9–16 Hz)^13^. The initial detections are used to calculate new search criteria for a second and final round, by taking the maximum likelihood estimates for the mean and standard deviation of each amplitude and frequency and excluding detections in the second search which are outside two standard deviations from the mean. For analyses distinguishing fast and slow spindles, the final count of spindling events is separated into slow and fast spindles using 13 Hz as a cut-off value (as in Schabus *et al*.^40^ and Hahn *et al*.^30^), that is, detections with frequency ≤ 13 Hz is categorized as slow, ≥13 Hz – fast. The frequency of slow and fast spindles overlaps at 13 Hz (see also histogram of population sigma frequency distribution for dogs on Fz and Cz in Iotchev *et al*.^11^). The mean amplitude of a recording was calculated as the average across spindle detections in that recording of the root-mean-square value for the signal segments corresponding to spindling events.

### Behavioral tests

#### Short-term memory task

**Figure 2:**
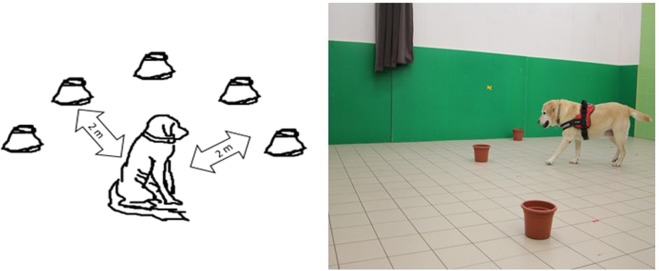
Arrangement short-term memory task (schematic, drawn by first author). The dog witnessed the baiting of one of the containers with a treat before being walked out of the room for 30 seconds (left). Subsequently the dog was returned to the starting position and was let free to find the baited container (right).

For the detailed protocol please see^23^. Briefly, five equally shaped, open containers were positioned in equal distance from each other in a semi-circular arrangement, resulting in each container being two meters away from the starting position of the dog (Fig. 2). A dog witnessed the baiting of one of the containers with a treat before being walked out of the room for 30 seconds. Subsequently the dog was returned to the starting position and was let free to find the baited container (see Piotti *et al*. for more details^23^). The task was repeated five times and each container was baited once in a random order. The measured variables were number of initially correct responses and number of wrong choices (since dogs had the possibility to visit any number of containers until they found the right location). However, in accordance with the conclusion of the original study^23^ we only use the number of initially correct choices in below analyses.

#### Reversal-learning

For the detailed protocol please see^49^. Briefly, dogs were first trained to either associate i) the spatial location (left or right) or ii) physical characteristics (large, black, rectangular plate vs. a small, white, round plate (plastic) of an object with the presence or absence of food (a dog was thus initially trained for either spatial location or physical characteristic based discrimination). The assignment to either reversal learning condition was counterbalanced between dogs and the conditions were switched between test and re-test to avoid carry-over effects.

In the spatial location condition the task relied on egocentric spatial coding (i.e., the animal could rely on the representation of the objects in space relative to its own body axes, such as left-right and front-back), specifically relying on learning (spatial function) and reversal-learning (executive function). For the physical characteristics condition, the tasks relied on visual learning and reversal learning (executive function). Both tasks also relied on visual discrimination-learning and reward and object approach-learning (learning domain). Previous findings suggested similar effects of age on location and size discrimination^49^; therefore, we expected younger dogs to perform better than old dogs in both tasks.

Dogs were presented with the positive and negative stimuli in consecutive trials; the stimuli were presented in a pre-determined pseudo-random order, with no more than two trials of the same type being presented consecutively. The experimenter put the plate on the floor and as soon as the plate was on the floor, the owner unleashed the dog. Owners were instructed to unleash the dogs exactly as the plate touched the floor, so to avoid biasing the dog’s behavior. The procedure is described in detail in Piotti *et al*.^47^ and follows criteria based on Kis *et al*.^50^. The paradigm was used to study discrimination learning before reversal-learning was tested. Importantly, dogs were included in a reversal-learning task only if they passed a threshold for learning the initial association (the longest latency for going to the correct location was shorter than any latency for going to the wrong location within the five vs. five most recent trials). The reversal-learning consisted of a maximum of 50 trials during which the rules were reversed (for instance, dogs who had to go left in the location-based association protocol, were now rewarded for going right). The present study makes use of the variable trial to criterion, that is, how many of the 50 trials were required for the dog to reach the threshold described for the preceding discrimination learning task. The reversal-learning part of the task was chosen instead of the discrimination learning part, due to its association with inhibition (as in suppression) as an underlying mechanism^51–53^, since it is hypothesized that spindles also support memory consolidation by suppressing irrelevant information^54^.

### Statistical Analysis

The structure of the data allowed us to investigate two questions. Firstly, whether a difference between the first and second recording of EEG is related to changes in performance between the first and second measure of short-term memory task and reversal-learning. Secondly, whether corresponding spindle and behavioral measures correlate when measured further apart than a day.

All analyses shared the same basic structure. Correlation matrices (Pearson) were calculated involving one of three behavioral tasks (the short-term memory task and each (starting) condition of the reversal-learning task were never tested at the same time for reasons explained below), three spindle-derived measures (frequency, density and amplitude) and age (in years) at the beginning of the experiment (prior to the first measurement). All analyses were conducted independently for both active electrodes (Fz, Cz) and spindle type (fast, slow). We did not include sex in analyses on the present sample, because more than 80% of the animals were neutered and sex differences in sleep spindles depend on sexual hormones in both humans and dogs^11,35–38^. The need to remove outliers was based on the standard scores for spindle amplitude. Subjects with values above or below 2.68 standard deviations from the group mean were excluded from the analyses (see Figure legends 3 and 4 for the total remaining sample size after this exclusion). The 2.68 threshold is derived from the quartile-based rule for detecting outliers, applied to the value likelihoods expected in a normal distribution (see for example http://www.cs.uni.edu/~campbell/stat/normfact.html). Based on previous observations in the dog^10,11^ and the human literature^3^, misleading extreme values are most likely to occur for spindle amplitudes.

### Analyses based on difference scores

Towards answering the first question, whether spindle measures and learning performance changed together, we calculated difference scores (2^nd^ – 1^st^ measurement) for each spindle measure and the performance on the short-term memory and reversal-learning tasks. Therefore, we had 6 difference scores (DS):(1) three EEG-derived DS for each density, amplitude and frequency of spindles, (2) short-term memory DS, (3) spatial location DS, (4) physical characteristics DS.

Analysis based on difference scores were preceded by testing for possible systematic changes in spindle frequency, density or amplitude from the 1^st^ to the 2^nd^ measurement, using paired-samples t-tests.

Separate correlation matrices were calculated for whether difference scores on the short-term memory task or trials to criterion on the reversal-learning task were included. For analyses based on the reversal-learning task we had to separately analyze each starting condition in accordance with earlier findings suggesting a strong effect of condition on the animals’ performance^47^, i.e. better performances were observed when the relevant cues were locations (as opposed to properties like color). Overall, analyses based on difference scores consisted for each electrode and spindle type of three correlation matrices – one across all dogs including only the difference scores for the short-term memory task as a behavioral measure and one for each starting condition on the reversal-learning task and including only difference scores on the reversal-learning task as a behavioral measure. In total 12 analyses (2 electrodes × 2 spindle types × 3 behavioral (sub-)tests) based on difference scores were performed.

### Analyses based on raw scores

Analyses based on raw (defined here as not obtained by calculating a difference as above) scores were controlled for the distance between corresponding behavioral and EEG measurements by using distance (in days) as a control variable in partial correlations.

For each electrode and spindle type, each series of recordings (first behavior and first EEG or second behavior and second EEG) were tested separately including only performance on the short-term memory task as a behavioral measure. Analysis for performance on the reversal-learning task, instead looked at each condition separately, but across all recordings (first and second behavior and EEG) obtained with the condition. We reasoned that the effect of condition observed previously^47^ would preclude the grouping of animals within a series of measurements, but instead it would be of interest how each condition affects spindle-performance associations across recordings obtained under the condition of interest. These resulted in four analyses based on raw scores per electrode and spindle type and 16 analyses ((2 electrodes × 2 spindle types) × 2 conditions (for reversal-learning) and/or × 2 measurements (for short-term memory)) in total.

### Correlations with age and between spindle-derived variables

Due to the overlap in variables included across correlation matrices, several comparisons were repeated under different sub-sets of the data. These concern correlations between spindle-derived measures and correlations with age. Due to this redundancy, we only consider correlations between spindle-measures and with age, from the largest sub-samples: 4 correlation matrices (2 ×2, for each electrode and spindle type) including performance on the short-term memory task, which were not separated by condition as those concerning the reversal-learning task.

### Corrections for multiple comparisons

Some of the comparisons that were repeated between correlation matrices were meaningfully different. For example, the correlation between difference scores for trials needed to criterion on the reversal learning task and spindle frequency was calculated for each spindle type, electrode, and starting condition (2 ×2 ×2) resulting in 8 independent multiple comparisons which we corrected using Bonferroni. In general, groups of tests to which Bonferroni was applied consisted of 8 comparisons (2 electrodes × 2 conditions/measurements (1^st^ or 2^nd^) × 2 spindle types), resulting in a Bonferroni significance threshold of 0.00625. Independence, which is required for Bonferroni^55^ was defined here either as samples not overlapping in subjects (dogs with different starting condition of the reversal learning task); samples not overlapping in recordings (recordings from different measurements i.e. 1^st^ or 2^nd^); or non-overlapping populations of spindles (fast versus slow). Below we report and plot findings which were significant at least prior correction and associated with performance on the learning and memory tasks for better overview. In a tabular overview (Table 2) we mark in addition which tests were significant under Bonferroni. For a complete overview of all correlation matrices see Supplementary Tables S1–S28.

**Table 2:** Overview of significant results. Bold indicates if comparisons were significant under Bonferroni.

association:	subpopulation:	direction:	nominal p-value:
trials to criterion (reversal learning) × fast spindle frequency	Fz, all recordings (raw scores), spatial location condition	positive	**0.003**
trials to criterion (reversal learning) × slow spindle frequency	Cz, difference scores, physical characteristics starting condition	positive	**0.004**
trials to criterion (reversal learning) × fast spindle frequency	Fz, difference scores, physical characteristics starting condition	positive	0.012
age × correct responses (short-term memory task)	Fz, raw scores, second series of measurements, fast spindle data set	negative	0.021
age × correct responses (short-term memory task)	Fz, raw scores, second series of measurements, slow spindle data set	negative	0.017
age × correct responses (short-term memory task)	Cz, raw scores, second series of measurements, fast spindle data set	negative	0.011
age × correct responses (short-term memory task)	Cz, raw scores, second series of measurements, slow spindle data set	negative	0.026
trials to criterion (reversal learning) × fast spindle frequency	Cz, difference scores, physical characteristics starting condition	positive	0.05

### Further control analyses

When similar correlations were tested twice (for different (starting) conditions of the reversal learning task or for the first and second series of measurements) and yielded different results we compared the correlation coefficients with an online calculator (https://www.psychometrica.de/correlation.html) employing a z-test method described in Eid, Gollwitzer and Schmidt (2011)^56^.

All analyses were performed in IBM SPSS, Release 22.0.0.0 (64-bit edition). Figures were made with GraphPad Prism.

## Results

### On Fz for fast spindles (13–16 Hz range)

#### Analyses based on difference scores

**Figure 3:**
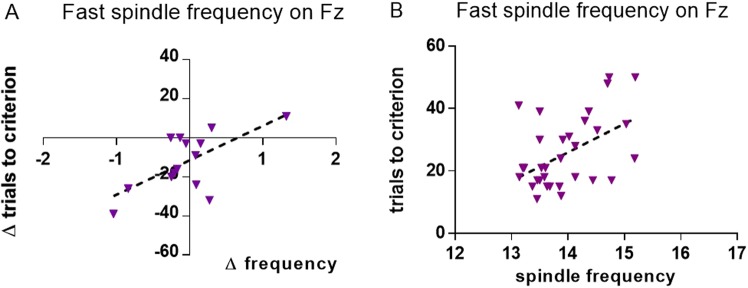
Change in fast spindle frequency versus change in trials to criterion on the reversal-learning task, 2^nd^ – 1^st^ measurement (N = 14) on Fz, for dogs who first attended the physical characteristics condition (**A**). Trials to criterion versus fast spindle frequency across all measurements for recordings obtained from the spatial location condition (N = 30, **B**).

In the reversal-learning test, for animals which started in the physical characteristics condition there was a negative correlation between the difference scores for trials needed to criterion and fast spindle frequency on Fz (r = −0.651, P = 0.012, Fig. 3A). This correlation was significantly different from the same test in animals which started in the spatial location condition (z = −1.756, P = 0.040) and displayed no significant association between the two variables (Supplementary Table S2).

#### Analyses based on raw scores

For the second series of measurements there was a significant negative correlation between age and correct responses on the short-term memory task (r = −0.392, P = 0.021). This correlation was not significantly different from the same test for the first series of measurements (z = 1.545, P = 0.061) which was not significant (see Supplementary Table S4).

In the reversal-learning task, across all recordings obtained in the spatial location condition, trials to criterion were positively correlated with fast spindle frequency (r = 0.507, P = 0.003, Fig. 3B). This correlation was not significantly different from the same test for recordings from the physical characteristics condition (z = 1.116, P = 0.132) which displayed no significant association between the two variables (Supplementary Table S7).

### On Fz for slow spindles (9–13 Hz range)

#### Analyses based on raw scores

For the second series of measurements, there was a significant negative correlation between age and correct responses in the short-term memory task (r = −0.38, P = 0.017). This correlation was not significantly different from the same test for the first series of measurements (z = 1.12, P = 0.131) which was not significant (see Supplementary Table S11).

### On Cz for fast spindles (13–16 Hz range)

#### Analyses based on difference scores

**Figure 4:**
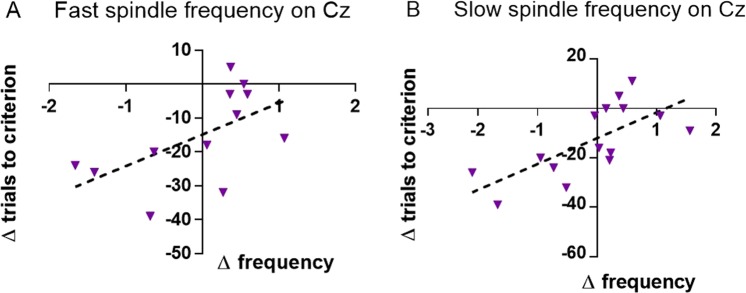
For animals which started with the physical characteristics condition: Change in trials needed to criterion on the reversal-learning task versus change in fast spindle frequency (N = 12, **A**) and change in slow spindle frequency (N = 15, **B**), 2^nd^ – 1^st^ measurement.

In the reversal-learning task, in animals which started with the physical characteristics condition, there was a positive correlation between the difference scores for fast spindle frequency and trials needed to criterion (r = 0.576, P = 0.050, Fig. 4A). This correlation was significantly different from the same test in animals which started with the spatial location condition (z = −2.234, P = 0.013) in which the association was not significant (Supplementary Table S16).

#### Analyses based on raw scores

In the second series of measurements a negative correlation was observed between age and correct responses on the short-term memory task (r = −0.415, P = 0.011). This correlation was not significantly different from the non-significant correlation obtained for the first series of measurements (z = 1.592, P = 0.056) (see Supplementary Table S18).

### On Cz for slow spindles (9–13 Hz range)

#### Analyses based on difference scores

For animals which started with the physical characteristics condition, there was a positive correlation between the difference scores for trials needed to criterion on the reversal-learning task and slow spindle frequency (r = 0.701, P = 0.004, Fig. 4B). This correlation was significantly different from the same test for animals which started with the spatial location condition (z = −1.832, P = 0.033) in which this association was not significant (Supplementary Table S23).

#### Analyses based on raw scores

For the second series of measurements there was a significant negative correlation between age and correct responses in the short-term memory task (r = −0.36, P = 0.026). This correlation was not significantly different from the one obtained for the first series of measurements (z = 1.075, P = 0.141) which was not significant (see Supplementary Table S25).

## Discussion

The question we set out to answer was whether some spindle characteristics in the dog are sufficiently stable biomarkers of learning and memory performance as to be valid even when they are temporally remote from measures of behavior.

Increases in fast spindle frequency over Fz and Cz, as well as slow spindle frequency over Cz correlated with worse performance on the reversal-learning task for dogs which began in the physical characteristics condition. Across recordings obtained in the spatial location condition on Fz the raw scores for fast spindle frequency and trials needed to criterion were also positively correlated. As a note of caution, however, only half of the correlations between spindle frequency and reversal learning remained significant under a Bonferroni correction (Table 2). A previous study in the dog reported that fast spindle frequency is higher in older subjects, and slow spindle frequency specifically higher in older males, but then exclusively over Cz^11^. Together, observations in this and the previous study suggest that in dogs, frequency increases are age-related changes in spindling that foreshadows cognitive decline. The here reported association between difference scores was specifically demonstrated for dogs who switched from the physical characteristics to the spatial location condition of the reversal-learning task. These correlations were significantly different from the non-significant correlations found for dogs who started in the spatial location condition, suggesting that condition sequence mattered for the observed effect. We suspect that a failure for this effect to surface in dogs who underwent the opposite condition switch is likely due to a cognitive switching cost, combined with the difference in difficulty between conditions. In favor of this interpretation, we also found that an association between fast spindle frequency and trials to criterion was significant across measurements in the easier spatial location condition, but not the more difficult^47^ physical characteristics condition. These two correlations did not differ significantly from each other, however. Moreover, there were also far fewer dogs that exhibited fast spindles and underwent the opposite condition switch (N = 7) thus this sample might also simply have been underpowered for the question at hand.

These results are somewhat surprising, as in humans age-related increases in frequency are not a frequent observation^29,33,48^ and are not always replicated^4^. However, Ktonas and colleagues, showed that dementia patients (albeit a small sample) displayed faster instantaneous spindle frequencies as compared to healthy controls^33^, which together with the here described findings makes spindling frequency the first marker of cognitive aging to be observed across species. Concerning possible mechanisms, two alternative scenarios could explain an age-related increase in spindling frequency as well as the here observed association of higher frequency with worse performance. By analogy, we could assume that similar to what is seen in the dopaminergic system, increased frequency is a compensatory response to cell-loss, exhibited by the surviving cells^57^. Alternatively, because the degree of suppression in the cortex can alter spindling frequency^58^, with the lowest frequencies exhibited during deep anesthesia, it can be reasoned that higher spindling frequencies reflect loss of cortical inhibition, which characterizes the aging process in humans^21,59^. Inhibition is also a crucial element in reversal-learning, via suppression of the original associations which ought to be reversed^51–53^. In favor of the former interpretation, we observed that on Cz, where increases in slow spindle frequency were correlated with worse reversal-learning performance, slow spindle density and frequency, as well as their difference scores, were also negatively correlated across several selections of data (Supplementary). However, fast spindle frequency and density (raw and difference scores) were instead positively correlated on both Fz and Cz. This effect also remained significant across different data-selections. It is not clear, yet, what the latter finding could tell about the underlying causal dynamics, but fast spindle density was previously found to increase with age in dogs^11^ and seems unique to the canine aging process. Since dogs, like humans, show a predominantly frontal decline in spindle amplitude^11,25,26^, it is possible that in dogs both frequency and density of fast spindles are augmented as a compensatory response.

Neither density (spindles/minute) nor amplitude, which if measured between acquisition and recall correlate with memory performance in the dog^10^ and human^10,18,32,39,60–62^, were associated with performance on either of the two tests, when behavior and EEG had been measured with distances greater than a day. Indeed, density is a likely unstable measure of spindle activity due to the influence of hormonal changes^35–37^ and diurnal experience^10,39–42^ on spindle occurrence, although in the present sample, of mostly neutered animals, only the latter applies. The link between spindle occurrence and post-sleep improvement on novel tasks is generally controversial. Although mechanistic work in animal models suggests a plausible explanation for such observations^17,63^ the largest (N > 900) study in humans to date could not confirm this notion^20^, moreover different factors appear to play a role in whether such an association is observed. These include timing relative to ripples and slow oscillation up-states^17–19^, as well as schema-compliance of the material to be encoded^16,17^. Amplitude was repeatedly found to correlate with at least IQ, but the strength of the association is modest^46^.

Important limitations of the present study are the high variation in time elapsed between corresponding measures of memory and EEG (for example 31.2 ± 48.2 days (M ± SD) for EEG versus short-term memory measurements). We can assume that due to developmental and age-related changes in spindle activity, confirmed also in the dog^11,64^, long time intervals could render allegedly corresponding EEG and behavioral measurements incomparable due to developmental changes. The interval between the two behavioral tests and EEG was an important control factor in our raw score analyses, because it was not fixed and varied from subject to subject. We therefore performed partial correlations controlling for distance (in days) between corresponding measurements of behavior and EEG.

A higher incidence of complete fast spindle absence in dogs was another problem. The overall data-loss for fast spindle analyses was between 29 and 30 dogs on Fz and Cz, respectively (approximately half of all subjects).

The distance between first and second measurements on each behavior and EEG was always at least 3 months. We deemed 3 months a considerable interval in dogs, because, for example, in many breeds reaching sexual maturity takes less than a year^65^. In support of this assumption, in the present study age and performance on the short-term memory task were associated only in the second series of measurements. As the sample of dogs for which EEG data was available is a sub-sample of the study in which an aging effect on the short-term memory task was originally described^23^ this asymmetry suggests that the 3 month interval between measurements captures a long-enough segment to observe age-related cognitive decline. At the end of the chosen interval the effect was strong enough to be observed also in the here reported sub-sample. Alternatively, sub-samples in which age with short-term memory were not correlated present Type II errors. The significant and non-significant correlations between memory and age were not significantly different from each other and the larger sample used in Piotti *et al*. suggests that performance on this task does decline with age^23^.

It is worth noticing that only reversal-learning, recognized in the literature as a fluid cognitive operation^51,53^, was associated with spindle characteristics in this sample. In the human literature, IQ is also associated with spindle characteristics independent of the distance between measurements of EEG and behavior^46^. Most measures of memory in humans and dogs, on the other hand, have been demonstrated to correlate with spindle occurrence (and occasionally amplitude or duration) only when spindles have been obtained from between acquisition and recall on the same day^10,18,32,39,60–62^. However, IQ-spindle associations concern amplitude and duration, and though well-replicated the effect sizes are small^46^.

Although a previous study suggests that age-related frequency increase in dogs is more pronounced over Cz^11^, no distinction had been made between the cognitive performance of individuals, whereas the aged dogs in the present sample were compared on two tasks of memory and learning. A pattern that emerges in comparison between both studies is that high fast spindle frequency in dogs (here on both Fz and Cz) is characteristic of both aging and performance decline, but this notion will require more empirical support. Currently the only similar study in humans relies on a fairly small sample^33^, while this first finding in the dog should be replicated in a more homogenous sample, with for example only one condition for reversal learning.

## Supplementary information

Supplementary.

Supplementary Dataset 1.

Supplementary Dataset 2.

Supplementary Dataset 3.
