# Supplementary material for: Possible association between spindle frequency and reversal-learning in aged family dogs
Source: Sci Rep. 2020 Apr 16;10:6505. doi: 10.1038/s41598-020-63573-9 (PMC7162895; doi:10.1038/s41598-020-63573-9)
Supplement: Supplementary file 1 — Supplementary. [file 41598_2020_63573_MOESM1_ESM.docx]

**Supplementary**

Possible association between spindle frequency and reversal-learning in aged family dogs

Ivaylo Borislavov Iotchev^a^, Dóra Szabó^a^, Anna Kis^b^, Enikő Kubinyi^a^

^a^ Department of Ethology, Eötvös Loránd University, Budapest, Hungary

^b^ Institute of Cognitive Neuroscience and Psychology, Research Centre for Natural Sciences, Budapest, Hungary

**Changes in spindle parameters across sessions (1^st^ versus 2^nd^ measurement)**

Spindle density, amplitude, or frequency did not change between the first and second measurement neither on Fz (fast spindles: all P > 0.2; slow spindles: all P > 0.05) nor on Cz (fast spindles: all P > 0.1; slow spindles: all P > 0.2).

**Correlation matrices**

Important abbreviations:

Difference scores:

1. diff_dens – spindles/minute 2^nd^ – 1^st^ measurement
2. diff_amp – spindle amplitude 2^nd^ – 1^st^ measurement
3. diff_freq – spindle (internal) frequency 2^nd^ – 1^st^ measurement
4. diff_correct – correct choices on the short-term memory task 2^nd^ – 1^st^ measurement
5. diff_reversal – trials to criterion on the reversal-learning task 2^nd^ – 1^st^ measurement

Raw scores:

1. dens – spindles/minute
2. amp – spindle amplitude
3. freq – spindle (internal) frequency
4. correct – correct choices on the short-term memory task
5. reversal_trial_nr – trials to criterion on the reversal-learning task

Correlation matrices for fast spindles on Fz

Difference scores

Short-term memory task

| **Correlations** | | | | | | |
| --- | --- | --- | --- | --- | --- | --- |
|  | | age | diff_dens | diff_amp | diff_freq | diff_correct |
| age | Pearson Correlation | 1 | .115 | .319 | .025 | .020 |
|  | Sig. (2-tailed) |  | .511 | .113 | .904 | .898 |
|  | N |  | 35 | 26 | 26 | 44 |
| diff_dens | Pearson Correlation |  | 1 | .138 | .604 | .186 |
|  | Sig. (2-tailed) |  |  | .502 | **.001** | .285 |
|  | N |  |  | 26 | 26 | 35 |
| diff_amp | Pearson Correlation |  |  | 1 | -.006 | .069 |
|  | Sig. (2-tailed) |  |  |  | .975 | .736 |
|  | N |  |  |  | 26 | 26 |
| diff_freq | Pearson Correlation |  |  |  | 1 | .169 |
|  | Sig. (2-tailed) |  |  |  |  | .410 |
|  | N |  |  |  |  | 26 |
| diff_correct | Pearson Correlation |  |  |  |  | 1 |

Table S1. Correlation matrix for age, all EEG difference scores and the difference scores for correct responses on the short-term memory task as the behavioral variable (diff_correct). For fast spindles on Fz. P-values which remained significant under Bonferroni are marked bold.

Reversal learning task, spatial location starting condition

| **Correlations** | | | | | | |
| --- | --- | --- | --- | --- | --- | --- |
|  | | age | diff_dens | diff_amp | diff_freq | diff_reversal |
| age | Pearson Correlation | 1 | .200 | .107 | .293 | .402 |
|  | Sig. (2-tailed) |  | .441 | .754 | .383 | .138 |
|  | N |  | 17 | 11 | 11 | 15 |
| diff_dens | Pearson Correlation |  | 1 | .279 | .807 | .019 |
|  | Sig. (2-tailed) |  |  | .405 | **.003** | .952 |
|  | N |  |  | 11 | 11 | 12 |
| diff_amp | Pearson Correlation |  |  | 1 | .044 | .216 |
|  | Sig. (2-tailed) |  |  |  | .898 | .643 |
|  | N |  |  |  | 11 | 7 |
| diff_freq | Pearson Correlation |  |  |  | 1 | -.243 |
|  | Sig. (2-tailed) |  |  |  |  | .599 |
|  | N |  |  |  |  | 7 |
| diff_reversal | Pearson Correlation |  |  |  |  | 1 |

Table S2. Correlation matrix for all EEG difference scores obtained under the spatial location starting condition and with difference scores for trials to criterion on the reversal-learning task (diff_reversal) as the behavioral variable. For fast spindles on Fz. P-values which remained significant under Bonferroni are marked bold.

Reversal learning task, physical characteristics starting condition

| **Correlations** | | | | | | |
| --- | --- | --- | --- | --- | --- | --- |
|  | | age | diff_dens | diff_amp | diff_freq | diff_reversal |
| age | Pearson Correlation | 1 | .076 | .368 | -.140 | .016 |
|  | Sig. (2-tailed) |  | .766 | .178 | .619 | .947 |
|  | N |  | 18 | 15 | 15 | 20 |
| diff_dens | Pearson Correlation |  | 1 | .059 | .210 | .113 |
|  | Sig. (2-tailed) |  |  | .835 | .453 | .666 |
|  | N |  |  | 15 | 15 | 17 |
| diff_amp | Pearson Correlation |  |  | 1 | -.027 | -.060 |
|  | Sig. (2-tailed) |  |  |  | .925 | .839 |
|  | N |  |  |  | 15 | 14 |
| diff_freq | Pearson Correlation |  |  |  | 1 | .651 |
|  | Sig. (2-tailed) |  |  |  |  | .012 |
|  | N |  |  |  |  | 14 |
| diff_reversal | Pearson Correlation |  |  |  |  | 1 |

Table S3. Correlation matrix for all EEG difference scores obtained under the physical characteristics starting condition and with the difference scores for trials to criterion on the reversal-learning task (diff_reversal) as the behavioral variable. For fast spindles on Fz. P-values which remained significant under a Bonferroni correction are marked bold.

Raw scores

Short-term memory task, first measurement

| **Correlations** | | | | | | | |
| --- | --- | --- | --- | --- | --- | --- | --- |
| Control Variables | | | age | dens | amp | freq | correct |
| distance | age | Correlation | 1 | .022 | -.076 | .129 | -.040 |
|  |  | Significance (2-tailed) | . | .887 | .625 | .405 | .796 |
|  |  | df |  | 42 | 42 | 42 | 42 |
|  | dens | Correlation |  | 1 | -.167 | .643 | -.044 |
|  |  | Significance (2-tailed) |  | . | .278 | **.000** | .779 |
|  |  | df |  |  | 42 | 42 | 42 |
|  | amp | Correlation |  |  | 1 | .032 | -.102 |
|  |  | Significance (2-tailed) |  |  | . | .834 | .511 |
|  |  | df |  |  |  | 42 | 42 |
|  | freq | Correlation |  |  |  | 1 | .173 |
|  |  | Significance (2-tailed) |  |  |  | . | .261 |
|  |  | df |  |  |  |  | 42 |
|  | correct | Correlation |  |  |  |  | 1 |
|  |  | Significance (2-tailed) |  |  |  |  | . |

Table S4. Correlation matrix for the first series of EEG measurements with correct responses on the short-term memory task (correct) as the behavioral variable. Correlations were corrected for distance (in days) between measurements of behavior and EEG. For fast spindles on Fz. P-values which remained significant under Bonferroni are marked bold.

Short-term memory task, second measurement

| **Correlations** | | | | | | | |
| --- | --- | --- | --- | --- | --- | --- | --- |
| Control Variables | | | age | dens | amp | freq | correct |
| distance | age | Correlation | 1 | .165 | -.104 | .218 | -.396 |
|  |  | Significance (2-tailed) | . | .351 | .560 | .216 | .021 |
|  |  | df |  | 32 | 32 | 32 | 32 |
|  | dens | Correlation |  | 1 | .122 | .531 | .183 |
|  |  | Significance (2-tailed) |  | . | .494 | **.001** | .300 |
|  |  | df |  |  | 32 | 32 | 32 |
|  | amp | Correlation |  |  | 1 | .093 | .023 |
|  |  | Significance (2-tailed) |  |  | . | .600 | .898 |
|  |  | df |  |  |  | 32 | 32 |
|  | freq | Correlation |  |  |  | 1 | .183 |
|  |  | Significance (2-tailed) |  |  |  | . | .299 |
|  |  | df |  |  |  |  | 32 |
|  | correct | Correlation |  |  |  |  | 1 |
|  |  | Significance (2-tailed) |  |  |  |  | . |

Table S5. Correlation matrix for the second series of EEG measurements with correct responses on the short-term memory task (correct) as the behavioral variable. Correlations were corrected for distance (in days) between measurements of behavior and EEG. For fast spindles on Fz. P-values which remained significant under Bonferroni are marked bold.

Reversal learning task, spatial location condition

| **Correlations** | | | | | | | |
| --- | --- | --- | --- | --- | --- | --- | --- |
| Control Variables | | | age | dens | amp | freq | reversal_trial_nr |
| distance | age | Correlation | 1 | .120 | .106 | .094 | .072 |
|  |  | Significance (2-tailed) | . | .511 | .563 | .609 | .694 |
|  |  | df |  | 30 | 30 | 30 | 30 |
|  | dens | Correlation |  | 1 | .124 | .673 | .317 |
|  |  | Significance (2-tailed) |  | . | .499 | **.000** | .077 |
|  |  | df |  |  | 30 | 30 | 30 |
|  | amp | Correlation |  |  | 1 | .308 | .253 |
|  |  | Significance (2-tailed) |  |  | . | .086 | .162 |
|  |  | df |  |  |  | 30 | 30 |
|  | freq | Correlation |  |  |  | 1 | .507 |
|  |  | Significance (2-tailed) |  |  |  | . | **.003** |
|  |  | df |  |  |  |  | 30 |
|  | reversal_trial_nr | Correlation |  |  |  |  | 1 |
|  |  | Significance (2-tailed) |  |  |  |  | . |

Table S6. Correlation matrix for analyses on the measurements obtained under the spatial location condition and with number of trials to criterion on the reversal-learning task (reversal_trial_nr) as the behavioral variable. Correlations were corrected for distance (in days) between measurements of behavior and EEG. For fast spindles on Fz. P-values which remained significant under Bonferroni are marked bold.

Reversal learning task, physical characteristics condition

| **Correlations** | | | | | | | |
| --- | --- | --- | --- | --- | --- | --- | --- |
| Control Variables | | | age | dens | amp | freq | reversal_trial_nr |
| distance1 | age | Correlation | 1 | .085 | -.314 | .249 | .117 |
|  |  | Significance (2-tailed) | . | .622 | .063 | .144 | .498 |
|  |  | df |  | 34 | 34 | 34 | 34 |
|  | dens | Correlation |  | 1 | -.104 | .619 | .232 |
|  |  | Significance (2-tailed) |  | . | .546 | **.000** | .173 |
|  |  | df |  |  | 34 | 34 | 34 |
|  | amp | Correlation |  |  | 1 | -.103 | -.208 |
|  |  | Significance (2-tailed) |  |  | . | .548 | .223 |
|  |  | df |  |  |  | 34 | 34 |
|  | freq | Correlation |  |  |  | 1 | .259 |
|  |  | Significance (2-tailed) |  |  |  | . | .127 |
|  |  | df |  |  |  |  | 34 |
|  | reversal_trial_nr | Correlation |  |  |  |  | 1 |
|  |  | Significance (2-tailed) |  |  |  |  | . |

Table S7. Correlation matrix for analyses on the measurements obtained under the physical characteristics condition and with number of trials to criterion on the reversal-learning task (reversal_trial_nr) as the behavioral variable. Correlations were corrected for distance (in days) between measurements of behavior and EEG. For fast spindles on Fz. P-values which remained significant under Bonferroni are marked bold.

Correlation matrices for slow spindles on Fz

Difference scores

Short-term memory task

| **Correlations** | | | | | | |
| --- | --- | --- | --- | --- | --- | --- |
|  | | age | diff_dens | diff_amp | diff_freq | diff_correct |
| age | Pearson Correlation | 1 | -.099 | -.093 | .175 | -.174 |
|  | Sig. (2-tailed) |  | .567 | .602 | .323 | .213 |
|  | N |  | 36 | 34 | 34 | 53 |
| diff_dens | Pearson Correlation |  | 1 | -.443 | -.074 | .125 |
|  | Sig. (2-tailed) |  |  | .009 | .678 | .466 |
|  | N |  |  | 34 | 34 | 36 |
| diff_amp | Pearson Correlation |  |  | 1 | -.095 | -.069 |
|  | Sig. (2-tailed) |  |  |  | .593 | .698 |
|  | N |  |  |  | 34 | 34 |
| diff_freq | Pearson Correlation |  |  |  | 1 | -.096 |
|  | Sig. (2-tailed) |  |  |  |  | .587 |
|  | N |  |  |  |  | 34 |
| diff_correct | Pearson Correlation |  |  |  |  | 1 |

Table S8. Correlation matrix for all EEG difference scores, using the difference scores for correct responses on the short-term memory task (correct) as the behavioral variable. For slow spindles on Fz. P-values which remained significant under Bonferroni are marked bold.

Reversal learning task, spatial location starting condition

| **Correlations** | | | | | | |
| --- | --- | --- | --- | --- | --- | --- |
|  | | age | diff_dens | diff_amp | diff_freq | diff_reversal |
| age | Pearson Correlation | 1 | .061 | -.238 | .513 | .217 |
|  | Sig. (2-tailed) |  | .810 | .357 | .035 | .457 |
|  | N |  | 18 | 17 | 17 | 14 |
| diff_dens | Pearson Correlation |  | 1 | -.633 | -.184 | .169 |
|  | Sig. (2-tailed) |  |  | .006 | .481 | .580 |
|  | N |  |  | 17 | 17 | 13 |
| diff_amp | Pearson Correlation |  |  | 1 | -.093 | -.239 |
|  | Sig. (2-tailed) |  |  |  | .723 | .455 |
|  | N |  |  |  | 17 | 12 |
| diff_freq | Pearson Correlation |  |  |  | 1 | .487 |
|  | Sig. (2-tailed) |  |  |  |  | .108 |
|  | N |  |  |  |  | 12 |
| diff_reversal | Pearson Correlation |  |  |  |  | 1 |

Table S9. Correlation matrix for all EEG difference scores obtained under the spatial location starting condition and with difference scores for trials to criterion on the reversal-learning task (diff_reversal) as the behavioral variable. For slow spindles on Fz. P-values which remained significant under Bonferroni are marked bold.

Reversal learning task, physical characteristics starting condition

| **Correlations** | | | | | | |
| --- | --- | --- | --- | --- | --- | --- |
|  | | age | diff_dens | diff_amp | diff_freq | diff_reversal |
| age | Pearson Correlation | 1 | -.112 | .320 | -.183 | .016 |
|  | Sig. (2-tailed) |  | .658 | .211 | .483 | .947 |
|  | N |  | 18 | 17 | 17 | 20 |
| diff_dens | Pearson Correlation |  | 1 | .040 | .153 | .022 |
|  | Sig. (2-tailed) |  |  | .878 | .559 | .934 |
|  | N |  |  | 17 | 17 | 17 |
| diff_amp | Pearson Correlation |  |  | 1 | -.124 | .298 |
|  | Sig. (2-tailed) |  |  |  | .636 | .262 |
|  | N |  |  |  | 17 | 16 |
| diff_freq | Pearson Correlation |  |  |  | 1 | -.112 |
|  | Sig. (2-tailed) |  |  |  |  | .680 |
|  | N |  |  |  |  | 16 |
| diff_reversal | Pearson Correlation |  |  |  |  | 1 |
|  | N |  |  |  |  |  |

Table S10. Correlation matrix for all EEG difference scores obtained under the physical characteristics starting condition and with difference scores for trials to criterion on the reversal-learning task (diff_reversal) as the behavioral variable. For slow spindles on Fz. P-values which remained significant under a Bonferroni correction are marked bold.

Raw scores

Short-term memory task, first measurement

| **Correlations** | | | | | | | |
| --- | --- | --- | --- | --- | --- | --- | --- |
| Control Variables | | | age | dens | amp | freq | correct |
| distance | age | Correlation | 1 | .199 | .001 | -.126 | -.148 |
|  |  | Significance (2-tailed) | . | .154 | .996 | .368 | .289 |
|  |  | df |  | 51 | 51 | 51 | 51 |
|  | dens | Correlation |  | 1 | -.451 | -.161 | -.028 |
|  |  | Significance (2-tailed) |  | . | **.001** | .248 | .843 |
|  |  | df |  |  | 51 | 51 | 51 |
|  | amp | Correlation |  |  | 1 | -.027 | -.003 |
|  |  | Significance (2-tailed) |  |  | . | .850 | .982 |
|  |  | df |  |  |  | 51 | 51 |
|  | freq | Correlation |  |  |  | 1 | .193 |
|  |  | Significance (2-tailed) |  |  |  | . | .166 |
|  |  | df |  |  |  |  | 51 |
|  | correct | Correlation |  |  |  |  | 1 |
|  |  | Significance (2-tailed) |  |  |  |  | . |

Table S11. Correlation matrix for the first series of measurements with correct responses on the short-term memory task (correct) as the behavioral variable. Correlations were corrected for distance (in days) between measurements of behavior and EEG. For slow spindles on Fz. P-values which remained significant Bonferroni are marked bold.

Short-term memory task, second measurement

| **Correlations** | | | | | | | |
| --- | --- | --- | --- | --- | --- | --- | --- |
| Control Variables | | | age | dens | amp | freq | correct |
| distance | age | Correlation | 1 | -.007 | .055 | .261 | -.380 |
|  |  | Significance (2-tailed) | . | .968 | .738 | .108 | .017 |
|  |  | df |  | 37 | 37 | 37 | 37 |
|  | dens | Correlation |  | 1 | -.508 | .220 | .123 |
|  |  | Significance (2-tailed) |  | . | **.001** | .179 | .455 |
|  |  | df |  |  | 37 | 37 | 37 |
|  | amp | Correlation |  |  | 1 | -.079 | .116 |
|  |  | Significance (2-tailed) |  |  | . | .634 | .481 |
|  |  | df |  |  |  | 37 | 37 |
|  | freq | Correlation |  |  |  | 1 | -.262 |
|  |  | Significance (2-tailed) |  |  |  | . | .107 |
|  |  | df |  |  |  |  | 37 |
|  | correct | Correlation |  |  |  |  | 1 |
|  |  | Significance (2-tailed) |  |  |  |  | . |

Table S12. Correlation matrix for the second series of measurements with correct responses on the short-term memory task (correct) as the behavioral variable. Correlations were corrected for distance (in days) between measurements of behavior and EEG. For slow spindles on Fz. P-values which remained significant under Bonferroni are marked bold.

Reversal learning task, spatial location condition

| **Correlations** | | | | | | | |
| --- | --- | --- | --- | --- | --- | --- | --- |
| Control Variables | | | age | dens | amp | freq | reversal_trial_nr |
| distance | age | Correlation | 1 | .062 | -.004 | -.158 | .024 |
|  |  | Significance (2-tailed) | . | .711 | .980 | .344 | .886 |
|  |  | df |  | 36 | 36 | 36 | 36 |
|  | dens | Correlation |  | 1 | -.551 | -.195 | -.183 |
|  |  | Significance (2-tailed) |  | . | **.000** | .240 | .270 |
|  |  | df |  |  | 36 | 36 | 36 |
|  | amp | Correlation |  |  | 1 | .042 | .191 |
|  |  | Significance (2-tailed) |  |  | . | .804 | .250 |
|  |  | df |  |  |  | 36 | 36 |
|  | freq | Correlation |  |  |  | 1 | .297 |
|  |  | Significance (2-tailed) |  |  |  | . | .070 |
|  |  | df |  |  |  |  | 36 |
|  | reversal_trial_nr | Correlation |  |  |  |  | 1 |
|  |  | Significance (2-tailed) |  |  |  |  | . |

Table S13. Correlation matrix for analyses on the measurements obtained under the spatial location condition and with number of trials to criterion on the reversal-learning task (reversal_trial_nr) as the behavioral variable. Correlations were corrected for distance (in days) between measurements of behavior and EEG. For slow spindles on Fz. P-values which remained significant under Bonferroni are marked bold.

Reversal learning task, physical characteristics condition

| **Correlations** | | | | | | | |
| --- | --- | --- | --- | --- | --- | --- | --- |
| Control Variables | | | age | dens | amp | freq | reversal_trial_nr |
| distance | age | Correlation | 1 | .309 | -.048 | .248 | .186 |
|  |  | Significance (2-tailed) | . | .052 | .767 | .123 | .250 |
|  |  | df |  | 38 | 38 | 38 | 38 |
|  | dens | Correlation |  | 1 | -.430 | .297 | .115 |
|  |  | Significance (2-tailed) |  | . | **.006** | .063 | .482 |
|  |  | df |  |  | 38 | 38 | 38 |
|  | amp | Correlation |  |  | 1 | -.253 | -.003 |
|  |  | Significance (2-tailed) |  |  | . | .115 | .985 |
|  |  | df |  |  |  | 38 | 38 |
|  | freq | Correlation |  |  |  | 1 | .275 |
|  |  | Significance (2-tailed) |  |  |  | . | .086 |
|  |  | df |  |  |  |  | 38 |
|  | reversal_trial_nr | Correlation |  |  |  |  | 1 |
|  |  | Significance (2-tailed) |  |  |  |  | . |

Table S14. Correlation matrix for analyses on the measurements obtained under the physical characteristics condition and with number of trials to criterion on the reversal-learning task (reversal_trial_nr) as the behavioral variable. Correlations were corrected for distance (in days) between measurements of behavior and EEG. For slow spindles on Fz. P-values which remained significant under Bonferroni are marked bold.

Correlation matrices for fast spindles on Cz

Difference scores

Short-term memory task

| **Correlations** | | | | | | |
| --- | --- | --- | --- | --- | --- | --- |
|  | | age | diff_dens | diff_amp | diff_freq | diff_correct |
| age | Pearson Correlation | 1 | .034 | -.112 | -.167 | .049 |
|  | Sig. (2-tailed) |  | .839 | .556 | .379 | .747 |
|  | N |  | 38 | 30 | 30 | 45 |
| diff_dens | Pearson Correlation |  | 1 | -.100 | .615 | .133 |
|  | Sig. (2-tailed) |  |  | .601 | **.000** | .427 |
|  | N |  |  | 30 | 30 | 38 |
| diff_amp | Pearson Correlation |  |  | 1 | -.133 | -.002 |
|  | Sig. (2-tailed) |  |  |  | .484 | .992 |
|  | N |  |  |  | 30 | 30 |
| diff_freq | Pearson Correlation |  |  |  | 1 | .077 |
|  | Sig. (2-tailed) |  |  |  |  | .686 |
|  | N |  |  |  |  | 30 |
| diff_correct | Pearson Correlation |  |  |  |  | 1 |

Table S15. Correlation matrix for all EEG difference scores, using the difference scores for correct responses on the short-term memory task (diff_correct) as the behavioral variable. For fast spindles on Cz. P-values which remained significant under Bonferroni are marked bold.

Reversal learning task, spatial location starting condition

| **Correlations** | | | | | | |
| --- | --- | --- | --- | --- | --- | --- |
|  | | age | diff_dens | diff_amp | diff_freq | diff_reversal |
| age | Pearson Correlation | 1 | .310 | -.205 | -.152 | .384 |
|  | Sig. (2-tailed) |  | .183 | .431 | .561 | .142 |
|  | N |  | 20 | 17 | 17 | 16 |
| diff_dens | Pearson Correlation |  | 1 | .066 | .539 | .044 |
|  | Sig. (2-tailed) |  |  | .802 | .026 | .877 |
|  | N |  |  | 17 | 17 | 15 |
| diff_amp | Pearson Correlation |  |  | 1 | -.044 | -.296 |
|  | Sig. (2-tailed) |  |  |  | .866 | .350 |
|  | N |  |  |  | 17 | 12 |
| diff_freq | Pearson Correlation |  |  |  | 1 | -.377 |
|  | Sig. (2-tailed) |  |  |  |  | .227 |
|  | N |  |  |  |  | 12 |
| diff_reversal | Pearson Correlation |  |  |  |  | 1 |

Table S16. Correlation matrix for all EEG difference scores obtained under the spatial location starting condition and with difference scores for trials to criterion on the reversal-learning task (diff_reversal) as the behavioral variable. For fast spindles on Cz. P-values which remained significant under Bonferroni are marked bold.

Reversal learning task, physical characteristics starting condition

| **Correlations** | | | | | | |
| --- | --- | --- | --- | --- | --- | --- |
|  | | age | diff_dens | diff_amp | diff_freq | diff_reversal |
| age | Pearson Correlation | 1 | -.128 | .110 | -.032 | .016 |
|  | Sig. (2-tailed) |  | .612 | .721 | .918 | .947 |
|  | N |  | 18 | 13 | 13 | 20 |
| diff_dens | Pearson Correlation |  | 1 | -.513 | .745 | .293 |
|  | Sig. (2-tailed) |  |  | .073 | **.003** | .253 |
|  | N |  |  | 13 | 13 | 17 |
| diff_amp | Pearson Correlation |  |  | 1 | -.527 | -.128 |
|  | Sig. (2-tailed) |  |  |  | .065 | .691 |
|  | N |  |  |  | 13 | 12 |
| diff_freq | Pearson Correlation |  |  |  | 1 | .576 |
|  | Sig. (2-tailed) |  |  |  |  | .050 |
|  | N |  |  |  |  | 12 |
| diff_reversal | Pearson Correlation |  |  |  |  | 1 |

Table S17. Correlation matrix for all EEG difference scores obtained under the physical characteristics starting condition and with difference scores for trials to criterion on the reversal-learning task (diff_reversal) as the behavioral variable. For fast spindles on Cz. P-values which remained significant under Bonferroni are marked bold.

Raw scores

Short-term memory task, first measurement

| **Correlations** | | | | | | | |
| --- | --- | --- | --- | --- | --- | --- | --- |
| Control Variables | | | age | dens | amp | freq | correct |
| distance | age | Correlation | 1 | .086 | .114 | .142 | -.064 |
|  |  | Significance (2-tailed) | . | .576 | .455 | .352 | .678 |
|  |  | df |  | 43 | 43 | 43 | 43 |
|  | dens | Correlation |  | 1 | -.126 | .622 | .033 |
|  |  | Significance (2-tailed) |  | . | .410 | **.000** | .830 |
|  |  | df |  |  | 43 | 43 | 43 |
|  | amp | Correlation |  |  | 1 | .146 | .092 |
|  |  | Significance (2-tailed) |  |  | . | .339 | .547 |
|  |  | df |  |  |  | 43 | 43 |
|  | freq | Correlation |  |  |  | 1 | -.118 |
|  |  | Significance (2-tailed) |  |  |  | . | .441 |
|  |  | df |  |  |  |  | 43 |
|  | correct | Correlation |  |  |  |  | 1 |
|  |  | Significance (2-tailed) |  |  |  |  | . |

Table S18. Correlation matrix for the first series of measurements with correct responses on the short-term memory task (correct) as the behavioral variable. Correlations were corrected for distance (in days) between measurements of behavior and EEG. For fast spindles on Cz. P-values which remained significant under Bonferroni are marked bold.

Short-term memory task, second measurement

| **Correlations** | | | | | | | |
| --- | --- | --- | --- | --- | --- | --- | --- |
| Control Variables | | | age | dens | amp | freq | correct |
| distance | age | Correlation | 1 | .123 | .072 | .071 | -.415 |
|  |  | Significance (2-tailed) | . | .467 | .672 | .675 | .011 |
|  |  | df |  | 35 | 35 | 35 | 35 |
|  | dens | Correlation |  | 1 | -.165 | .580 | .201 |
|  |  | Significance (2-tailed) |  | . | .329 | **.000** | .233 |
|  |  | df |  |  | 35 | 35 | 35 |
|  | amp | Correlation |  |  | 1 | .081 | .015 |
|  |  | Significance (2-tailed) |  |  | . | .634 | .931 |
|  |  | df |  |  |  | 35 | 35 |
|  | freq | Correlation |  |  |  | 1 | .117 |
|  |  | Significance (2-tailed) |  |  |  | . | .492 |
|  |  | df |  |  |  |  | 35 |
|  | correct | Correlation |  |  |  |  | 1 |
|  |  | Significance (2-tailed) |  |  |  |  | . |

Table S19. Correlation matrix for the second series of measurements with correct responses on the short-term memory task (correct) as the behavioral variable. Correlations were corrected for distance (in days) between measurements of behavior and EEG. For fast spindles on Cz. P-values which remained significant under Bonferroni are marked bold.

Reversal learning task, spatial location condition

| **Correlations** | | | | | | | |
| --- | --- | --- | --- | --- | --- | --- | --- |
| Control Variables | | | age | dens | amp | freq | reversal_trial_nr |
| distance | age | Correlation | 1 | -.054 | .229 | .128 | -.150 |
|  |  | Significance (2-tailed) | . | .764 | .200 | .479 | .404 |
|  |  | df |  | 31 | 31 | 31 | 31 |
|  | dens | Correlation |  | 1 | -.112 | .657 | -.039 |
|  |  | Significance (2-tailed) |  | . | .536 | **.000** | .831 |
|  |  | df |  |  | 31 | 31 | 31 |
|  | amp | Correlation |  |  | 1 | .131 | -.262 |
|  |  | Significance (2-tailed) |  |  | . | .467 | .141 |
|  |  | df |  |  |  | 31 | 31 |
|  | freq | Correlation |  |  |  | 1 | .014 |
|  |  | Significance (2-tailed) |  |  |  | . | .937 |
|  |  | df |  |  |  |  | 31 |
|  | reversal_trial_nr | Correlation |  |  |  |  | 1 |
|  |  | Significance (2-tailed) |  |  |  |  | . |

Table S20. Correlation matrix for analyses on the measurements obtained under the spatial location condition and with number of trials to criterion on the reversal-learning task (reversal_trial_nr) as the behavioral variable. Correlations were corrected for distance (in days) between measurements of behavior and EEG. For fast spindles on Cz. P-values which remained significant under Bonferroni are marked bold.

Reversal learning task, physical characteristics condition

| **Correlations** | | | | | | | |
| --- | --- | --- | --- | --- | --- | --- | --- |
| Control Variables | | | age | dens | amp | freq | reversal_trial_nr |
| distance | age | Correlation | 1 | .238 | .106 | .124 | .076 |
|  |  | Significance (2-tailed) | . | .163 | .539 | .472 | .661 |
|  |  | df |  | 34 | 34 | 34 | 34 |
|  | dens | Correlation |  | 1 | -.167 | .550 | .316 |
|  |  | Significance (2-tailed) |  | . | .332 | **.001** | .060 |
|  |  | df |  |  | 34 | 34 | 34 |
|  | amp | Correlation |  |  | 1 | .059 | .152 |
|  |  | Significance (2-tailed) |  |  | . | .730 | .377 |
|  |  | df |  |  |  | 34 | 34 |
|  | freq | Correlation |  |  |  | 1 | .127 |
|  |  | Significance (2-tailed) |  |  |  | . | .460 |
|  |  | df |  |  |  |  | 34 |
|  | reversal_trial_nr | Correlation |  |  |  |  | 1 |
|  |  | Significance (2-tailed) |  |  |  |  | . |

Table S21. Correlation matrix for analyses on the measurements obtained under the physical characteristics condition and with number of trials to criterion on the reversal-learning task (reversal_trial_nr) as the behavioral variable. Correlations were corrected for distance (in days) between measurements of behavior and EEG. For fast spindles on Cz. P-values which remained significant under Bonferroni are marked bold.

Correlation matrices for slow spindles on Cz

Difference scores

Short-term memory task

| **Correlations** | | | | | | |
| --- | --- | --- | --- | --- | --- | --- |
|  | | age | diff_dens | diff_amp | diff_freq | diff_correct |
| age | Pearson Correlation | 1 | .031 | -.054 | .100 | -.010 |
|  | Sig. (2-tailed) |  | .853 | .759 | .568 | .949 |
|  | N |  | 37 | 35 | 35 | 44 |
| diff_dens | Pearson Correlation |  | 1 | -.341 | -.466 | .025 |
|  | Sig. (2-tailed) |  |  | .045 | **.005** | .882 |
|  | N |  |  | 35 | 35 | 37 |
| diff_amp | Pearson Correlation |  |  | 1 | .108 | .037 |
|  | Sig. (2-tailed) |  |  |  | .536 | .834 |
|  | N |  |  |  | 35 | 35 |
| diff_freq | Pearson Correlation |  |  |  | 1 | .060 |
|  | Sig. (2-tailed) |  |  |  |  | .730 |
|  | N |  |  |  |  | 35 |
| diff_correct | Pearson Correlation |  |  |  |  | 1 |

Table S22. Correlation matrix for all EEG difference scores, using the difference scores for correct responses on the short-term memory task (diff_correct) as the behavioral variable. For slow spindles on Cz. P-values which remained significant under Bonferroni are marked bold.

Reversal learning, spatial location starting condition

| **Correlations** | | | | | | |
| --- | --- | --- | --- | --- | --- | --- |
|  | | age | diff_dens | diff_amp | diff_freq | diff_reversal |
| age | Pearson Correlation | 1 | .044 | -.087 | .325 | .299 |
|  | Sig. (2-tailed) |  | .855 | .722 | .174 | .279 |
|  | N |  | 20 | 19 | 19 | 15 |
| diff_dens | Pearson Correlation |  | 1 | -.482 | -.681 | .260 |
|  | Sig. (2-tailed) |  |  | .037 | **.001** | .369 |
|  | N |  |  | 19 | 19 | 14 |
| diff_amp | Pearson Correlation |  |  | 1 | .274 | .016 |
|  | Sig. (2-tailed) |  |  |  | .257 | .956 |
|  | N |  |  |  | 19 | 14 |
| diff_freq | Pearson Correlation |  |  |  | 1 | .104 |
|  | Sig. (2-tailed) |  |  |  |  | .724 |
|  | N |  |  |  |  | 14 |
| diff_reversal | Pearson Correlation |  |  |  |  | 1 |

Table S23. Correlation matrix for all EEG difference scores obtained under the spatial location starting condition and with difference scores for trials to criterion on the reversal-learning task (diff_reversal) as the behavioral variable. For slow spindles on Cz. P-values which remained significant under Bonferroni are marked bold.

Reversal learning, physical characteristics starting condition

| **Correlations** | | | | | | |
| --- | --- | --- | --- | --- | --- | --- |
|  | | age | diff_dens | diff_amp | diff_freq | diff_reversal |
| age | Pearson Correlation | 1 | .071 | -.164 | -.118 | .074 |
|  | Sig. (2-tailed) |  | .786 | .544 | .663 | .763 |
|  | N |  | 17 | 16 | 16 | 19 |
| diff_dens | Pearson Correlation |  | 1 | -.137 | -.285 | -.016 |
|  | Sig. (2-tailed) |  |  | .612 | .285 | .953 |
|  | N |  |  | 16 | 16 | 16 |
| diff_amp | Pearson Correlation |  |  | 1 | -.119 | .161 |
|  | Sig. (2-tailed) |  |  |  | .660 | .566 |
|  | N |  |  |  | 16 | 15 |
| diff_freq | Pearson Correlation |  |  |  | 1 | .701 |
|  | Sig. (2-tailed) |  |  |  |  | **.004** |
|  | N |  |  |  |  | 15 |
| diff_reversal | Pearson Correlation |  |  |  |  | 1 |

Table S24. Correlation matrix for all EEG difference scores obtained under the physical characteristics starting condition and with difference scores for trials to criterion on the reversal-learning task (diff_reversal) as the behavioral variable. For slow spindles on Cz. P-values which remained significant under Bonferroni are marked bold.

Raw scores

Short-term memory task, first measurement

| **Correlations** | | | | | | | |
| --- | --- | --- | --- | --- | --- | --- | --- |
| Control Variables | | | age | dens | amp | freq | correct |
| distance | age | Correlation | 1 | -.141 | .402 | -.106 | -.134 |
|  |  | Significance (2-tailed) | . | .308 | **.003** | .443 | .334 |
|  |  | df |  | 52 | 52 | 52 | 52 |
|  | dens | Correlation |  | 1 | -.322 | -.325 | .116 |
|  |  | Significance (2-tailed) |  | . | .018 | .017 | .405 |
|  |  | df |  |  | 52 | 52 | 52 |
|  | amp | Correlation |  |  | 1 | -.335 | -.065 |
|  |  | Significance (2-tailed) |  |  | . | .013 | .638 |
|  |  | df |  |  |  | 52 | 52 |
|  | freq | Correlation |  |  |  | 1 | .158 |
|  |  | Significance (2-tailed) |  |  |  | . | .255 |
|  |  | df |  |  |  |  | 52 |
|  | correct | Correlation |  |  |  |  | 1 |
|  |  | Significance (2-tailed) |  |  |  |  | . |

Table S25. Correlation matrix for the first series of measurements with correct responses on the short-term memory task (correct) as the behavioral variable. Correlations were corrected for distance (in days) between measurements of behavior and EEG. For slow spindles on Cz. P-values which remained significant under Bonferroni are marked bold.

Short-term memory task, second measurement

| **Correlations** | | | | | | | |
| --- | --- | --- | --- | --- | --- | --- | --- |
| Control Variables | | | age | dens | amp | freq | correct |
| distance | age | Correlation | 1 | -.016 | .022 | .182 | -.360 |
|  |  | Significance (2-tailed) | . | .926 | .895 | .275 | .026 |
|  |  | df |  | 36 | 36 | 36 | 36 |
|  | dens | Correlation |  | 1 | -.312 | -.359 | -.083 |
|  |  | Significance (2-tailed) |  | . | .057 | .027 | .619 |
|  |  | df |  |  | 36 | 36 | 36 |
|  | amp | Correlation |  |  | 1 | .050 | -.031 |
|  |  | Significance (2-tailed) |  |  | . | .767 | .855 |
|  |  | df |  |  |  | 36 | 36 |
|  | freq | Correlation |  |  |  | 1 | .129 |
|  |  | Significance (2-tailed) |  |  |  | . | .439 |
|  |  | df |  |  |  |  | 36 |
|  | correct | Correlation |  |  |  |  | 1 |
|  |  | Significance (2-tailed) |  |  |  |  | . |

Table S26. Correlation matrix for the second series of measurements with correct responses on the short-term memory task as the behavioral variable (correct). Correlations were corrected for distance (in days) between measurements of behavior and EEG. For slow spindles on Cz. P-values which remained significant under Bonferroni are marked bold.

Reversal learning, spatial location condition

| **Correlations** | | | | | | | |
| --- | --- | --- | --- | --- | --- | --- | --- |
| Control Variables | | | age | dens | amp | freq | reversal_trial_nr |
| distance | age | Correlation | 1 | -.092 | .275 | -.021 | .032 |
|  |  | Significance (2-tailed) | . | .589 | .100 | .901 | .851 |
|  |  | df |  | 35 | 35 | 35 | 35 |
|  | dens | Correlation |  | 1 | -.174 | -.395 | -.157 |
|  |  | Significance (2-tailed) |  | . | .302 | .016 | .353 |
|  |  | df |  |  | 35 | 35 | 35 |
|  | amp | Correlation |  |  | 1 | -.294 | .179 |
|  |  | Significance (2-tailed) |  |  | . | .078 | .290 |
|  |  | df |  |  |  | 35 | 35 |
|  | freq | Correlation |  |  |  | 1 | .153 |
|  |  | Significance (2-tailed) |  |  |  | . | .367 |
|  |  | df |  |  |  |  | 35 |
|  | reversal_trial_nr | Correlation |  |  |  |  | 1 |
|  |  | Significance (2-tailed) |  |  |  |  | . |

Table S27. Correlation matrix for analyses on the measurements obtained under the spatial location condition and with number of trials to criterion on the reversal-learning task (reversal_trial_nr) as the behavioral variable. Correlations were corrected for distance (in days) between measurements of behavior and EEG. For slow spindles on Cz. P-values which remained significant under Bonferroni are marked bold.

Reversal learning, physical characteristics condition

| **Correlations** | | | | | | | |
| --- | --- | --- | --- | --- | --- | --- | --- |
| Control Variables | | | age | dens | amp | freq | reversal_trial_nr |
| distance | age | Correlation | 1 | .044 | .185 | .120 | .215 |
|  |  | Significance (2-tailed) | . | .783 | .241 | .450 | .172 |
|  |  | df |  | 40 | 40 | 40 | 40 |
|  | dens | Correlation |  | 1 | -.468 | -.277 | .104 |
|  |  | Significance (2-tailed) |  | . | **.002** | .075 | .511 |
|  |  | df |  |  | 40 | 40 | 40 |
|  | amp | Correlation |  |  | 1 | -.110 | .068 |
|  |  | Significance (2-tailed) |  |  | . | .489 | .667 |
|  |  | df |  |  |  | 40 | 40 |
|  | freq | Correlation |  |  |  | 1 | .109 |
|  |  | Significance (2-tailed) |  |  |  | . | .492 |
|  |  | df |  |  |  |  | 40 |
|  | reversal_trial_nr | Correlation |  |  |  |  | 1 |
|  |  | Significance (2-tailed) |  |  |  |  | . |

Table S28. Correlation matrix for analyses on the measurements obtained under the physical characteristics condition and with number of trials to criterion on the reversal-learning task (reversal_trial_nr) as the behavioral variable. For slow spindles on Cz. P-values which remained significant under Bonferroni are marked bold.
